# The variation of insulin like growth factor 2 maker is associated with growth traits in Thai native (Kradon) pigs

**DOI:** 10.5713/ab.22.0431

**Published:** 2023-05-04

**Authors:** Kessara Ampaporn, Rattikan Suwannasing, Pitchayanipa Phongphanich, Supanon Tunim, Monchai Duangjinda

**Affiliations:** 1 Department of Animal Science, Faculty of Natural Resources, Rajamangala University of Technology Isan Sakon Nakon Campus, Sakon Nakon 47160, Thailand; 2 Animal Production and Management Division, Faculty of Natural Resources, Prince of Songkla University, Songkla 90112, Thailand; 3 Department of Animal Science, Faculty of Agriculture, Khon Kaen University, Khon Kaen 40002, Thailand

**Keywords:** Insulin Like Growth Factor 2 (*IGF2*) Gene, Kradon Pig, Melanocortin-4 Receptor (*MC4R*) Gene, Thai Native Pig

## Abstract

**Objective:**

This study was conducted to investigate polymorphisms of the melanocortin-4 receptor (*MC4R*) and insulin like growth factor 2 (*IGF2*) genes and to evaluate the growth traits affected by such polymorphisms in Thai native (Kradon) pigs.

**Methods:**

Blood samples and productive data from 91 Kradon pigs were collected. DNA was extracted and quantified, the *IGF2* and *MC4R* genes were amplified, and the polymerase chain reaction (PCR) produces were digested using the PCR-restriction fragment length polymorphism (PCR-RFLP) technique. Genotyping was performed, and the association between genotypes and growth traits on the birth and weaning weights were evaluated.

**Results:**

The IGF2 intron7 g.162G>C variations in Kradon pigs were found in three genotypes: i) GG, ii) GC, and iii) CC. The GG genotype frequency was the highest followed by the GC and CC genotypes. The frequencies of the G and C alleles were 0.703 and 0.297, respectively. The *MC4R* genotype was found in only one genotype (GG). The *IGF2* gene pattern was not associated with birth weight traits, whereas the *IGF2* gene pattern was related to the weaning weight trait in Kradon pigs. Pigs with the CC and GC genotypes had higher weaning weights than ones with the GG genotype (p<0.001).

**Conclusion:**

Thai native Kradon pigs with the CC and GC genotypes of the *IGF2* gene have higher weaning weights than pigs with the GG genotype.

## INTRODUCTION

Four distinct breeds of Thai native pigs, Hainan, Rad, Puang, and Kwai, are found in Thai farms [1]. The Rad or Kradon pigs, found in the Northeast of Thailand [2], have the smallest body size among Thai native pigs and are entirely black in color, have a fine meat texture, small bones, and delicious meat [3]. Generally, they are raised in the backyards in the Northeast Thailand and are usually fed with residues of plant products found around houses and crops. Kradon pigs have long been associated with rural peoples’ culture as most of this population raises Kradon pigs for household consumption during festivals, such as New Year and Songkran. Kradon pigs are adapted to their environment and have delicious meat even after consuming low quality feeds. However, Thai native pigs are decreasing in number, due to a slow-growth rate and poor carcasses quality that has high abdominal fat. Therefore, the selection for growth and red meat production is necessary for increased production and to conserve the genetic diversity of Thai native pigs.

The insulin like growth factor 2 (*IGF2*) gene (or somatomedin A) is located on chromosome 2 (SSC2). This gene spans 30 kbp and consists of nine exons, eight introns, and four promoters. *IGF2* exons 7, 8, and 9 encode prepro *IGF2* protein [4], and exons 1–6 are non-coding [5]. The *IGF2* gene is one of the potentially important genetic markers for the growth and carcass traits of pigs [6]. *IGF2* concentration is associated with body weight in growing pigs [7]. The substitution at the *IGF2* intron7 g.162G>C results in increased growth and body size of pigs [8,9] but a decrease in body length (BL) and back fat (BF). According our previous study [10], the association of *IGF2* gene with back fat depth at 10^t^ rib and last rib in Thai native pig was revealed. The Canadian Center for Swine Improvement (CCSI) cooperates with GENTEC, Belgium to investigate the effects of the *IGF2* gene in a Canadian wild pig population. It was found that the *IGF2* gene controls red meat production in wild pigs. Thus, the *IGF2* gene can be used to both increase and decrease the amount of red meat in wild pigs. This report will be useful in creating commercial wild pig breeds, which will be capable of producing wild pigs that are uniform in terms of red meat production. The *IGF2* gene might be used to select Thai native pigs to improve growth performance.

The melanocortin-4 receptor (*MC4R*) gene in pigs is primarily responsible for energy homeostasis. It is located on chromosome 1 and has a total sequence length of 18 kp with 1 exon. At position c.746G>A (Asp298Asn), the transition from aspartic (Asp) to asparagine (Asn) was found to be associated with an increase in growth rate (average daily gain [ADG]) and feed intake (FI) in pigs [11–13]. The pigs without the *MC4R* mutation showed thinner BF traits, lower ADG, and less FI. The mutation of the *MC4R* gene was used as a DNA marker to improve the growth traits of pigs.

Hence, the *IGF2* and *MC4R* genes were suitable candidate genes for growth trait studies. The objectives of this study were to investigate the polymorphisms of the *IGF2* and *MC4R* genes and evaluate whether these polymorphisms affect the growth traits in Kradon pigs.

## MATERIALS AND METHODS

### Animal care

This experiment was reviewed and approved by the Institutional Animal Care and Use Committee of Rajamangala University of Technology Isan (No.48/2564).

### Animals and data collections

A random population from a total of 91 blood samples of Kradon pigs (39 castrated males and 52 females) were collected from the swine farm unit of the Department of Animal Science, Faculty of Natural Resources, Rajamangala University of Technology Isan, Sakon Nakhon Campus. All pigs were raised under an open-house system in cement pens of 2×4 m and were handled with identical procedures. The pigs received clean water from a nipple drinker and were fed with a diet of not less than 20% crude protein (CP). Their birth and weaning weights were recorded. Blood samples were collected from the jugular vein by needles No.18G×1 in for a volume of 5 to 10 mL of collected blood and added to a 15 mL tube containing 0.5 M ethylenediaminetetraacetic acid. These samples were centrifuged at 4,200 rpm for 5 min to precipitate the white blood cells and then stored at −4°C until they were used for DNA extraction.

### DNA extraction and quantification

The buffy coat or white blood cells was prepared from 50 μL of whole blood added to 1.5 mL microtubes after which 1,000 μL 0.9% NaCl was added to the tubes, and then mixed and vortexed for 5 to 10 s. After centrifugation at 10,000 rpm for 5 min, the supernatant was discarded. Genomic DNA was extracted by a guanidine hydrochloride protocol modified procedure developed by Goodwin et al [14]. Fifty microliters of white blood cells were divided into 1.5 mL microtubes, and then 70 μL 20% SDS, 50 μL 7.5 M Na-acetate, 25 μL 1% Proteinase K, and 625 μL 5 M Guanidine HCl were added to the tubes and mixed by flipping the microtubes up and down 2 to 3 times and vortexed for 5 to 10 s. They were then incubated overnight at 65°C in a waterbath. These microtubes were centrifuged at 10,000 rpm for 5 min after which 500 μL of supernatant was aspirated into the new tube. Five-hundred microliters of cold absolute isopropanol was then added to this mixture, The microtubes were flipped up and down, and DNA precipitation was noted. The microtubes were then centrifugated at 10,000 rpm for 5 min after which the supernatant was discarded while the DNA precipitate was isolated. The DNA was washed by adding 500 μL of 75% ethanol and then centrifuged at 10,000 rpm for 1 min after which the supernatant was discarded twice, while the DNA was isolated. The DNA was dried at room temperature for 30 to 60 min after which 25 μL of TE buffer was added, and the DNA was incubated in a waterbath at 37°C overnight to dissolve the DNA. One microliter of the DNA sample was used to determine the DNA quality using a NanoDrop2000 spectrophotometer (Nano Drop Thermo Scientific, Wilmington, DE, USA) at 260 and 280 nm wavelengths. The DNA concentration was adjusted to 50 ng/μL and stored at −20°C for use in subsequent amplification.

### PCR-RFLP and genotyping

The primers for the *IGF2* and *MC4R* genes were designed from published sequences. The *IGF2* primer sequence has been reported by Vykoukalová et al [15] and the *MC4R* primer sequence was reported by Dvorakova et al [16]. The *IGF2* and *MC4R* fragments were amplified using polymerase chain reaction (PCR), 10 μL contained 4.1 μL distill water, 1 μL dNTP (1 mM; Fermentas, Hanover, MD, USA), 0.8 μL MgCl_2_ (25 mM), 1 μL 10× PCR buffer, 1 μL of 3 μM each primer (forward and reverse primer), 0.1 μL 5 U Taq DNA polymerase (MBI Fermentas, USA), and 1 μL 50 ng genomics DNA. Primers and are listed in Table 1. A T-Professional Standard Thermo cycler (Biometra GmbH, Gottingen, Germany) was used for amplification under specific conditions: i) 95°C for 3 min, ii) 30 cycles at 95°C for 30 s, iii) annealing for 30 s and 72°C for 45 s, and iv) a final extension at 72°C for 5 min. The products were evaluated using a 2% agarose gel electrophoresis.

The PCR products of *IGF2* and *MC4R* genes were digested in a total volume of 10 μL, containing 6.7 μL of distilled water, 2 μL PCR products, 1 μL cut smart buffer, and 0.3 μL each of restriction enzymes, and the reactions were digested overnight at 37°C for *Bcn*I, and 65°C for *Taq*I (New England Biolabs, Ipswich, MA, USA). The digested PCR products of *IGF2* and *MC4R* were separated on 2% agarose gel electrophoresis at 100 V for 30 min, and RFLP fragments were visualized using Gel Star (Lonza, Rockland, ME, USA). The *IGF2* gene pattern were three genotypes pattern with GG, GC, and CC. GG genotype DNA banding sizes were 308 and 28 bp, GC genotypes were 308, 208, 100, and 28 bp, and CC genotype pattern were 208, 100, and 28 bp. Additionally, *MC4R* genotyping with three patterns demonstrated the presence of GG, AG, and AA. The GG pattern was represented with 156 and 70 bp, GA genotype was 226, 156 and 70 bp, and AA genotype was 226 bp.

### Data analysis

The genotype patterns of *IGF2* and *MC4R* genes were used for calculating the gene and genotype frequencies, observed heterozygosity (H_O_), expected heterozygosity (H_E_), and unbiased expected heterozygosity (H_U_) following Nei’s method [17]. The polymorphic information content (PIC) was assessed according to the method by Botstein et al [18]. The Hardy–Weinberg Equilibrium of the *IGF2* and *M4CR* genes was tested using the GENEPOP software (version 4.2) available online: http://genepop.curtin.edu.au/. The investigation of the association between the polymorphism of genotype pattern and both birth and weaning weight traits was carried out with the general linear model procedure using the SAS procedure (Statistical Analysis System 9.0; SAS Institute, Cary, NC, USA) by the following statistical linear model for birth weight trait according to Y_ijk_ = μ+Sex_i_+D_j_+G_k_+ɛ_ijk_, and weaning weight trait according to Y_ijk_ = μ+Sex_i_+D_j_+W_k_+G_l_+ɛ_ijk_ in which Y_ijk_ represents the phenotypic values; μ is the overall mean; Sex_i_ represents the sex effect; D_j_ is the maternal effect; W_k_ is the covariate of birth weight effect; G_k_ and G_l_ are genotype effects; and ɛ_ijk_ represents the random error.

## RESULTS

### Polymorphism of *IGF2* and *MC4R* markers in Kradon pigs

**Figure 1:**
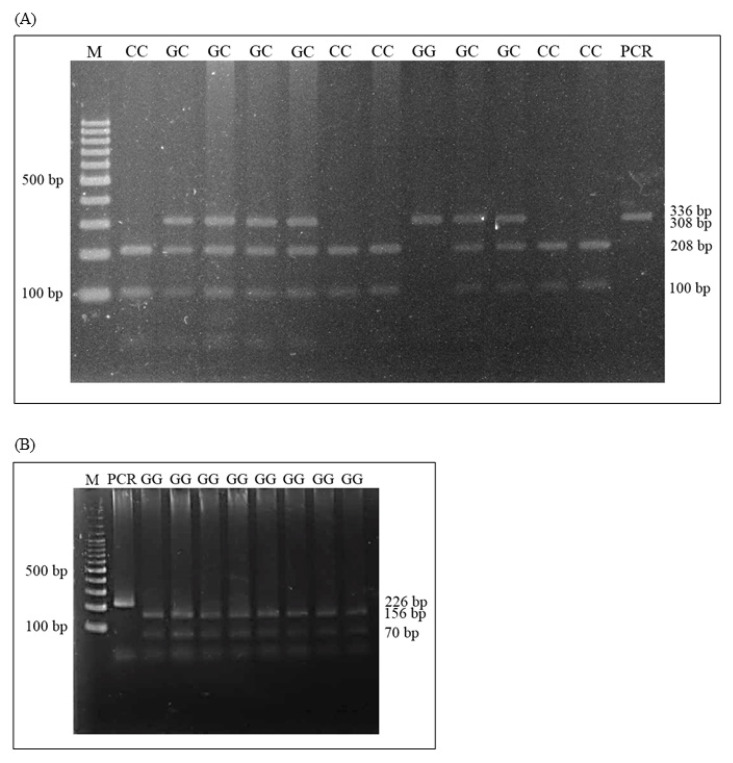
PCR-RFLP patterns: (A) *IGF2/Bcn*I; (B) *MC4R/Taq*I genes in Thai Kradon pigs. PCR-RFLP, polymerase chain reaction-restriction fragment length polymorphism; *IGF2*, insulin like growth factor 2; *MC4R*, melanocortin-4 receptor. M = 100 bp DNA ladder; PCR = PCR product; GG, GC, and CC = genotypes.

Genotype patterns of the genes were studied from the amplified gene fragments that were digested with the restriction enzymes, and it was found that the size of the *IGF2* and *MC4R* gene fragments matched the size of the reference reports. *IGF2* gene variations in Kradon pigs found with the three genotypes were GG, GC, and CC (Figure 1A). The polymorphism of *MC4R* was found in only the GG genotype with fragment sizes of 156 and 70 bp (Figure 1B).

Our study of polymorphism of the *IGF2* gene analyzed the allele and genotype frequencies, and it was found that the GG genotype frequency was the highest (0.516) followed by the GC genotype frequency (0.374), and that the CC genotype frequency was the lowest (0.110) as shown in Table 2 with an allele frequency G and C of 0.703 and 0.297, respectively.

The HWE analysis is reported in Table 2. The results showed the *IGF2* genotype followed the null hypothesis that stated that Kradon pigs might not be affected by gene forces because this initial population was maintained in a relatively closed area without a systematic breeding program. Thus, migration and selection factors may not significantly affect the HWE of the *IGF2* gene. In contrast, *MC4R* gene could not undergo HWE analysis because it is a non-diverse allele with only GG genotype.

### Association of *IGF2* genotypes with birth weight and weaning weight traits

An evaluation of the *MC4R* gene was not feasible because only one genotype pattern was found. Therefore, the association between the genotype pattern and both their birth and weaning weight traits could not be analyzed. However, the association of *IGF2* with birth and weaning weight traits was analyzed. The results of the study found that the genotype pattern was not associated with birth weight traits (p>0.05). Regardless of the genotype of the Thai native pigs, no influence on the birth weight of piglets was found. However, a correlation between the *IGF2* gene pattern and weaning weight traits was found (Table 3). Pigs with the CC genotype had the highest weaning weight, and pigs with the GG genotype had the lowest (p<0.001).

## DISCUSSION

The genotypic pattern of the *IGF2* gene, that is the CC, GC, and GG genotypes, found in the Thai native pig is the same as in commercial pig breeds such as Large White, Landrace, Duroc, Pietrain, and Yorkshire [19,20]. The Thai native pigs had GG genotype frequencies (0.516) higher than the GC and CC genotypes (0.374 and 0.110, respectively), and G allele frequencies (0.703) higher than C allele frequencies (0.297). This report was consistent with the report of Klomtong et al [19] who mentioned that the genotypic frequencies of the *IGF2* genes with GG, GC, and CC genotypes were 0.570, 0.270, and 0.160, respectively. In contrast, with regard to the genotype frequency of the *IGF2* gene in commercial pigs, the CC genotype has a higher genotype frequency than the GG genotype [15,19]. It can be concluded that the genetic structure of the *IGF2* gene is different in Thai native pigs versus commercial pigs. This difference may be an important factor for Thai native pigs to have lower growth rates than commercial pigs. Therefore, if the genetic structure of the *IGF2* gene were manipulated in the Kradon pigs so that it has a higher CC genotype, it may help the Thai native pigs have a better growth rate and an improved carcass quality than the present ones.

In this study, *IGF2* gene variations were found to be related to the weaning weight characteristics of the Kradon pigs, which may be caused by hepatic *IGF2* expression in the pigs. Clark et al [21] delineated the effects of the *IGF2* mutation on the expression of myogenic gene during prenatal and postnatal growth. Male and female offspring were evaluated at birth (0 d) and weaning (21 d), and it was found that at 21 d hepatic, the *IGF2* expression was greater (p = 0.01). The increased *IGF2* expression may contribute to an increase in the muscle mass of pigs [22]. The partial knockout of zinc finger BED-type containing 6 (*ZBED6*) could affect the secretion of *IGF2* in pig liver [23]. The interaction between the *ZBED6* and the *IGF2* locus plays a prominent role in up-regulating postnatal growth of skeletal muscle and internal organs; kidney, liver, and heart [24]. Reports of the *IGF2* gene expression and its effect on fatty acid composition in different pig genetic backgrounds are available. Twenty-five percent of the gene expression indicated significant associations between *IGF2* polymorphism and arachidonic, hexadecenoic, oleic, linoleic, and α-linoleic fatty acids and the monounsaturated fatty acid/polyunsaturated fatty acid (MUFA/PUFA) ratio measured in BF [25].

The *IGF2* gene has also been reported to be associated with economically important traits of various pig breeds, such as the genotypic pattern of the *IGF2* gene in increased red meat and fat thickness in pigs [8,20]. Braunschweig [26] reported that the *IGF2* gene is expressed in organs, such as the brain, liver, muscles, and kidneys. Therefore, it is possible that the *IGF2* gene associations may be found at all stages of the maturation of pigs during the fetal and postnatal stages [27]. This association may depend on the breed of the pigs or the size of the population used in the study. Duroc pigs, especially, have high frequencies of alleles at the *IGF2* intron 3 g.3072G>A loci that favor lean production and stress resistance [28].

The polymorphisms of the *IGF2* gene have the potential to be used for marker-assisted selection of growth traits in Kradon pigs. The selection and mating of animals with CC and GC genotypes can help improve the growth performance of Kradon pigs. However, to more precisely assess the effects of *IGF2* polymorphism on growth and meat production, it would be necessary to increase the population size.

This work differs from that reported by Chaweewan et al [29] who reported that the *MC4R* genotypes, AA, GA and GG genotypes were found in the hybrid pig breeds, Pak Chong 1–5. This difference may be due to a decrease in the population size of Kradon pigs in Thailand, which may be a factor affecting the gene diversity of the *MC4R* gene in Kradon pigs when compared with Large White, Landrace, Duroc, Pietrain, and other pig breeds, developed by the Department of Livestock Development of Thailand such as Pak Chong 1–5. In this study, the number of *MC4R* gene variants differed from those reported by Fan et al [30] and Szyndler-Nędza et al [31] who found three *MC4R* genotypes in pigs. Piórkowska et al [32] suggested that the A allele should be selected to produce improvements in litter size and fertility in the dam line and to increase the frequency of the G allele in the sire line to obtain pigs with high red meat content.

The *MC4R* gene is not only related to the growth characteristics of pigs but is also associated with an increase in fat thickness [33] and feed intake in pigs [12]. The missense mutation of the *MC4R* gene with alternating G and A bases resulted in the amino acid at position 298 shifting from the aspartic (Asp) codon, GAU, and GAC to an asparagine (Asn) coded AAU and AAC codon whose A allele was found to correlate with feed intake, whereas the G allele was associated with high red meat content in commercial and hybrid pigs [29]. However, in this study, only a single genotypic pattern of the *MC4R* gene was found in the Kradon pig. Therefore, to study the association of *MC4R* genes with economically important traits of Kradon pigs, the number of samples should be increased to have a variety of genes.

## CONCLUSION

The genotype pattern of the *IGF2* gene in Kradon pigs were CC, GC, and GG genotypes. The CC genotypes of the *IGF2* gene had a higher weaning weight than pigs with the GC and GG genotypes. On the other hand, the *MC4R* had a GG genotype in Kradon pigs.

## Figures and Tables

**Table 1:** Accession number, primer sequences, Tm and PCR product sizes of *IGF2* and *MC4R* markers

Genes	Accession no./SNPs	Primer sequence	Tm (°C)	Size (bp)
*IGF2*	X56094.162G>C (Intron7)	F: 5′-CACAGCAGGTGCTCCATCGG-3′	62	336
		R: 5′-GACAGGCTGTCATCCTGTGG-3′		
*MC4R*	AF087937: c.746G>A	F: 5′-TAC CCT GAC CAT CTT GAT TG- 3′	60	226
		R: 5′-ATAGCAACAGATGATCTCTTTG-3′		

PCR, polymerase chain reaction; *IGF2*, insulin like growth factor 2; *MC4R*, melanocortin-4 receptor; SNPs, single nucleotide polymorphism.

**Table 2:** Genotype and allele frequencies, H_O_, H_E_, H_U_, PIC, and chi-square test (*x*^2^) of *IGF2* and *MC4R* genes in Thai Kradon pigs

Genes	N	Frequency		H_O_	H_E_	H_U_	PIC	HWE	p-value

		Genotype	Allele						
*IGF2*	10	0.516 (GG)	0.703 (G)	0.374	0.417	0.420	0.330	0.998	0.607
	34	0.374 (GC)	0.297 (C)						
	47	0.110 (CC)							
*MC4R*	0	1.000 (GG)	1.000 (G)						
	0	0.000 (GA)	0.000 (A)	0.000	0.000	0.000	0.000	0.000	<0.001
	91	0.000 (AA)							

N, number of samples; H_O_, observed heterozygosity; H_E_, expected heterozygosity; H_U_, unbiased expected heterozygosity; PIC, polymorphic information content; *IGF2*, insulin like growth factor 2; *MC4R*, melanocortin-4 receptor; HWE, Hardy-Weinberg equilibrium by the x^2^-test (α = 0.05).

**Table 3:** Least square means and standard error for birth weight and weaning weight by *IGF2* genotypes in Thai Kradon pigs

Traits	*IGF2* genotypes			p-value

	GG	GC	CC	
Birth weight (kg)	0.414±0.021	0.439±0.027	0.501±0.048	0.297
Weaning weight (kg)	4.992±0.210^c^	6.013±0.275^b^	7.522±0.497^a^	<0.0001

*IGF2*, insulin like growth factor 2.

a–c Mean values with different superscript letters within a row denote significant (p<0.05) differences between groups.
